# TAVI-in-TAVI in a patient with morquio syndrome: a case report

**DOI:** 10.1093/ehjcr/ytaf662

**Published:** 2026-01-19

**Authors:** Diana Xiao Lan Chin, Gianantonio De Michele, Davide Cristofani, Francesco De Felice

**Affiliations:** Department of Cardiology, Interventional Cardiology Unit, San Camillo Hospital, Circ.ne Gianicolense 87, Rome 00152, Italy; Department of Translational Medical Sciences; Division of Cardiology, University of Campania Luigi Vanvitelli, A.O.R.N. ‘Sant'Anna & San Sebastiano’, Caserta 81100, Italy; Department of Cardiology, Anesthesiology and Intensive Care Unit, San Camillo Hospital, Rome 00152, Italy; Department of Cardiology, Interventional Cardiology Unit, San Camillo Hospital, Circ.ne Gianicolense 87, Rome 00152, Italy

**Keywords:** Morquio Syndrome, TAVI-in-TAVI, Bioprosthetic valve failure, Self-expanding transcatheter valve, Aortic stenosis, Case report

## Abstract

**Background:**

Morquio syndrome (MPS IV) is a rare multi-systemic disorder with significant cardiovascular implications, including early-onset valvular disease. Due to the improved life expectancy, these patients could require complex interventional solutions such as TAVI-in-TAVI procedure.

**Case summary:**

We present the first reported case of a 65-year-old woman with Morquio syndrome undergoing a TAVI-in-TAVI procedure for structural degeneration of a prior transcatheter bioprosthetic valve. The procedure was technically challenging due to complex thoracic anatomy, small aortic annulus, and intermediate-risk of coronary obstruction. A self-expanding Evolut FX + valve were successfully implanted with favourable haemodynamic outcomes and no major complications.

**Conclusion:**

This case highlights the feasibility and importance of individualized planning in complex redo-TAVI interventions in patients with rare congenital disorders.

Learning points

- Unfavourable features increase the risk of bioprosthesis degeneration in patients with Morquio syndrome undergoing transcatheter aortic valve implantation (TAVI).
- Proper Heart Team discussion and pre-procedural planning can dramatically reduce the risk of suboptimal results and coronary occlusion.

## Introduction

Morquio Syndrome or Mucopolysaccharidosis Type IV (MPS IV) is an autosomal recessive disorder caused by the lack of N-acetylgalactosamine-6-sulfate leading to glycosaminoglycans (GAGs) accumulation in various tissues in the body. Cardiopulmonary complications include airway narrowing, restrictive lung function, pulmonary hypertension, mitral and aortic valve stenoses and regurgitation together with arterial wall accumulation of mycopolysaccharide.^1^ We report the first documented case of TAVI-in-TAVI in a patient with Morquio syndrome and prior transcatheter valve dysfunction.

## Summary figure

**Figure:**
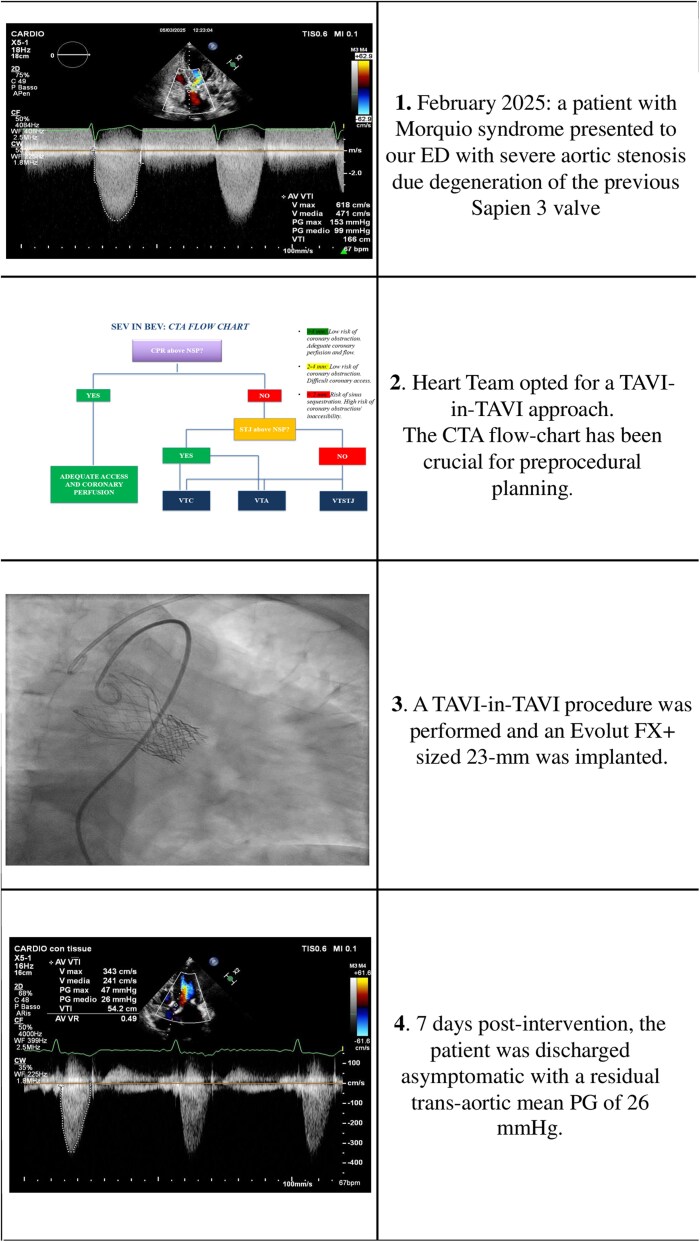


## Case presentation

**Figure 1:**
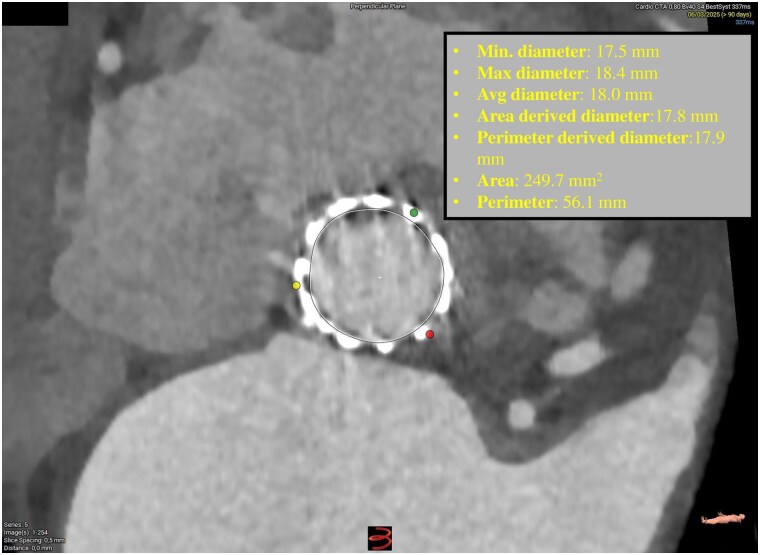
Aortic anulus dimensions at computed tomography. Avg, average.

**Figure 2:**
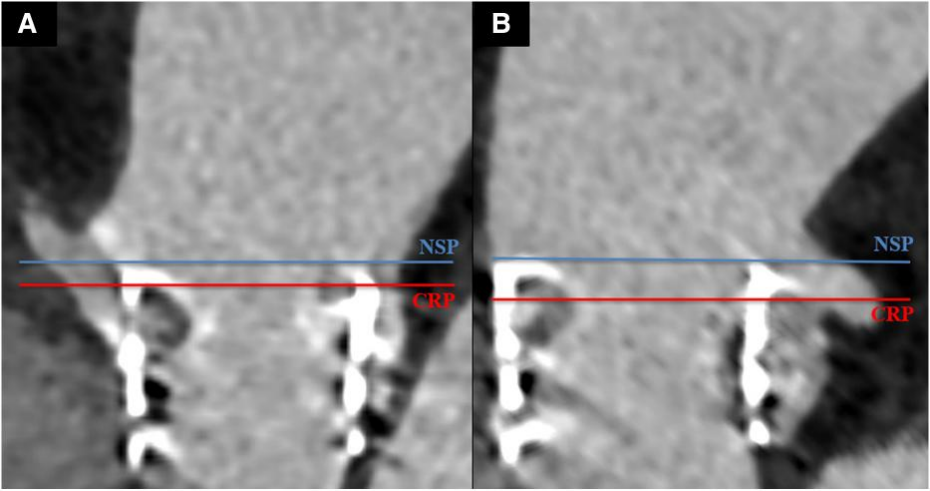
At computed tomography, relationship between coronary risk plane (CRP) and neo skirt plane (NSP) for right coronary artery (*A*) and left coronary artery (*B*).

**Figure 3:**
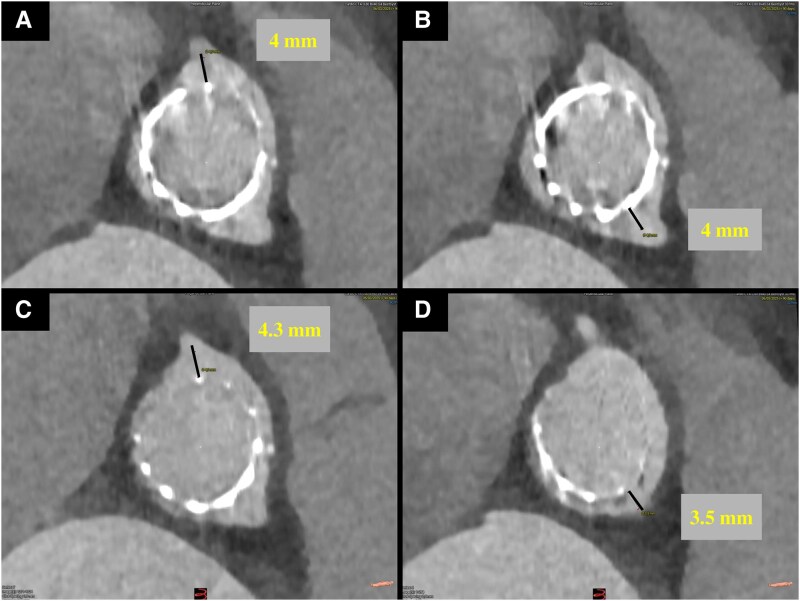
(*A*) Valve to RCA distance (defined as the measurement from the valve frame to the level of the right coronary ostium distance); (*B*) Valve to LCA distance (defined as the measurement from the valve frame to the level of the left coronary ostium distance); (*C*) Valve to Aorta for RCA (defined as the measurement from valve frame to aortic wall distance at the level of neo skirt plane for the right coronary artery); (*D*) Valve to Aorta for LCA (defined as the measurement from valve frame to aortic wall distance at the level of neo skirt plane for the left coronary artery).

**Figure 4:**
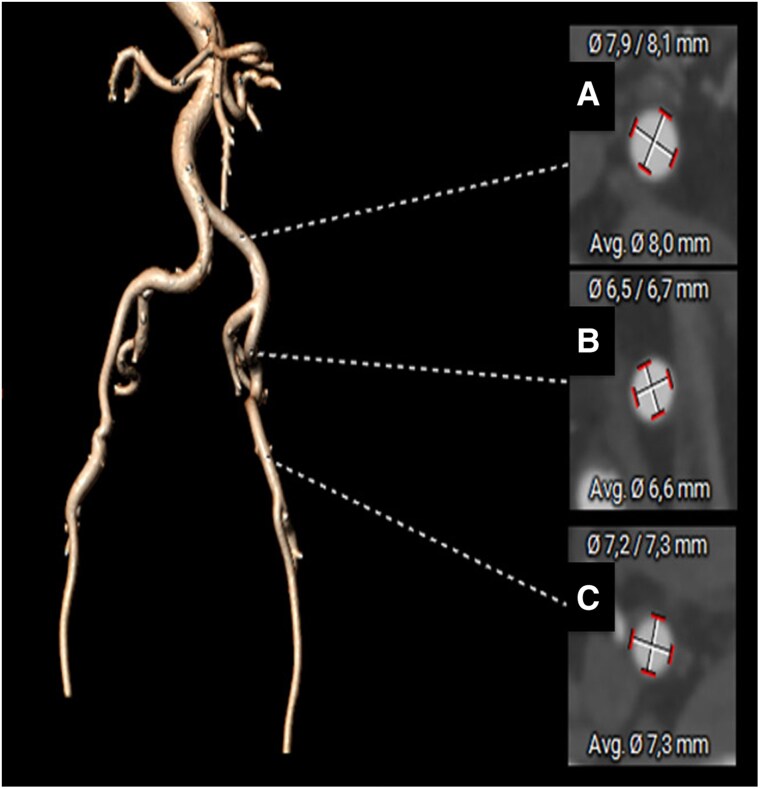
At computed tomography, iliofemoral arteries reconstruction. (*A*) Dimensions at the left common iliac artery level; (*B*) dimensions at the left external iliac artery level; (*C*) dimensions at the common left femoral artery level.

A 65-year-old woman with Morquio syndrome presented to the emergency department (ED) with dyspnoea and signs of heart failure (HF). At age of 57, she underwent TAVI, using a 20 mm Sapien3 valve (Edwards Lifesciences, Inc., Irvine, California) due to high surgical risk. The initial procedure was complicated by dissection of the right iliac and common femoral arteries, which was managed conservatively. Post-procedural echocardiogram showed a mean transaortic pressure gradient (PG) of 30 mmHg, and the patient remained mildly symptomatic for 6 years. At the age of 63, worsening HF symptoms and signs prompted an aortic valvuloplasty using 20- and 21-mm aortic balloons, which lowered the mean transaortic PG to 12 mmHg. Two years later, she presented to our ED with progressive dyspnoea. Clinical evaluation revealed a body surface area (BSA) of 1.36 m² (height 140 cm, weight 50 kg), a loud systolic aortic murmur, bilateral fine crepitations on lung auscultation, and peripheral oedema. Electrocardiogram showed signs of left ventricular hypertrophy, and blood tests indicated elevated N-terminal pro-Brain Natriuretic Perptide (NT pro-BNP) at 6000 pg/mL, with normal renal function and complete blood count. Transthoracic echocardiography revealed degeneration of the transcatheter heart valve (THV) with severe aortic stenosis (mean PG 107 mmHg, peak velocity 4.9 m/sec) and moderate aortic regurgitation. The left ventricle showed concentric hypertrophy with preserved ejection fraction, and no other significant valvular disease was identified. The Heart Team deemed the patient inoperable due to the complexity of thoracic anatomy and anaesthetic challenges associated with Morquio syndrome. Pre-procedural computed tomography (CT) analysis was performed using 3mensio software (3mensio Medical Imaging BV, Utrecht, NL) and confirmed feasibility of transfemoral access. The aortic annulus measured 234 mm² in area, with a minimum diameter of 17.2 mm and a perimeter of 54.4 mm (*Figure 1*). The neo-skirt plane (NSP), was located above the coronary risk plane (CRP) (*Figure 2*), with a valve to coronary distance (VTC) of 4 mm for both the coronaries and a valve to aorta measurement (VTA) of 3.5 mm for the left and 4.3 mm for the right coronary artery (*Figure 3*). The right iliofemoral arteries showed residual signs of dissection, whereas the left iliofemoral arteries measured minimal diameters of 6.5 × 6.7 mm (*Figure 4*), without calcification, albeit some tortuosity. Consequently, the left common femoral artery was chosen as the principal access site. Considering the small annulus and high residual gradient after the first TAVI, a 23 mm self-expanding Evolut FX + (Medtronic) valve was chosen for its supra-annular deployment characteristics. The procedure was performed under conscious sedation with anaesthesiology support. The left radial artery served as ancillary access. Micropuncture of the left femoral artery and pre-implantation of two Proglides were performed. A 14Fr sheath was inserted, and a Safari XS wire was advanced across the degenerated valve. Predilation with an 18 × 40 mm balloon optimized haemodynamics before valve deployment. Initial results showed moderate paravalvular leak and a residual transaortic PG of 40 mmHg. Post-dilation with a 20 × 40 mm balloon successfully reduced the invasive peak-to-peak gradient to 15 mmHg, with no angiographic evidence of residual leak. The patient was discharged after 7 days on single antiplatelet therapy. At 30-day follow-up, she remained asymptomatic. Echocardiography showed a mean PG of 25 mmHg and mild perivalvular leak.

## Discussion

**Figure 5:**
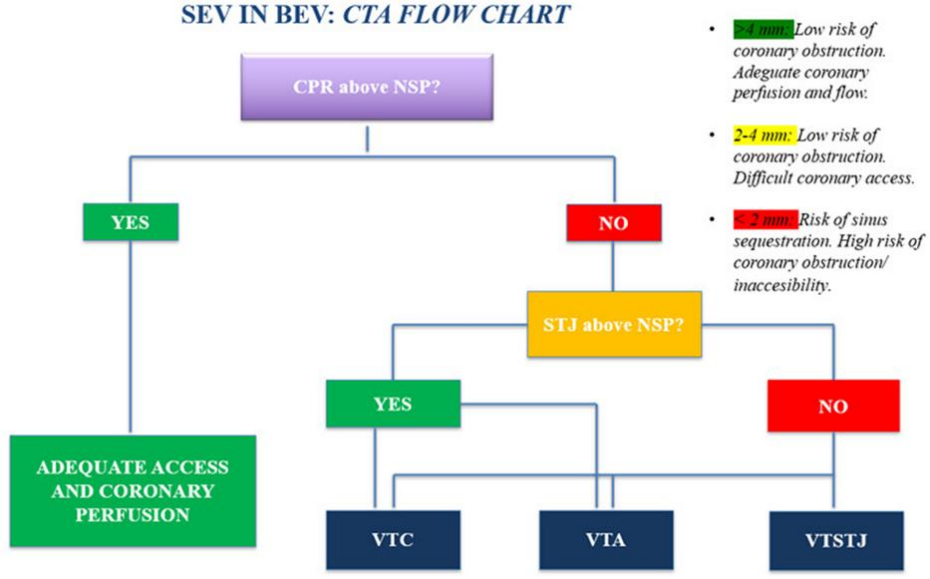
Self-expandable valve in balloon-expandable valve BEV, imaging flow chart; CPR, coronary risk plane; NSP, neoskirt risk plane; STJ, sinotubular junction plane; VTA, valve to aorta; VTC, valve to coronary; VTSTJ, valve to STJ.

Morquio syndrome is characterized by multi-systemic involvement. Historically, life expectancy of affected individuals was limited to the third decade.^2^ However, advances in medical care and multidisciplinary management have enabled many to live into their 70 s.^3^ This claim is coherent with data reported by Perez RH *et al*. who described patients with Morquio syndrome living until 74 years.^4^ With increased longevity, these individuals are now presenting with severe aortic stenosis at younger ages. The first reported case of aortic valve replacement in patients with Morquio syndrome was in 2006^5^ and only recently the first TAVI in a patient with Morquio syndrome was published.^6^ Surgical valve replacement is often contraindicated due to complex anatomy, making TAVI the preferred approach. Nonetheless, structural degeneration of bioprosthetic valves necessitates reintervention, increasingly involving TAVI-in-TAVI procedures. The Heart Team is mandatory to decide between redo-TAVI and TAVI-explantation. The EXPLANTORREDO-TAVR registry analyzed over 66 000 TAVI cases, 396 of them (0.59%) underwent reintervention: TAVI-explant (181, 46.4%) or redo-TAVI (215, 54.3%). Both at 30 days (13.6% vs. 3.4%; *P* < 0.001) and at 1-year follow-up (32.4% vs. 15.4%; *P* = 0.001), the TAVI-explant cohort had a higher mortality rate than the redo-TAVI group.^7^ Despite these results, coronary access impairment or obstruction remains a major concern in TAVI-in-TAVI,^8^ especially when implanting a self-expanding valve into a balloon-expandable frame. During the procedure, the leaflets of the original valve may be displaced upward and jailed between the two THVs frames, forming a neo-skirt that obstructs flow in the sinuses of Valsalva and limits coronary access. The spatial relationship between the neo-skirt and coronary ostia is critical. When the NSP is located above the CRP, access may be compromised depending on the height of the sinotubular junction (STJP). In such scenarios, VTC and VTA measurements become decisive. VTC or VTA values above 4 mm generally predict low risk of coronary obstruction, whereas measurements between 2 and 4 mm indicate intermediate-risk, and <2 mm signify high risk of sinus sequestration and coronary obstruction^9^ (*Figure 5*). In our patient, the VTC and VTA values of 3.5 to 4.3 mm placed her in the intermediate-risk category. With meticulous pre-procedural planning and appropriate device selection, the risk was deemed acceptable. Another important consideration is the risk of prosthesis-patient mismatch (PPM), particularly relevant in our patient who has a small annulus and low BSA. PPM has been associated with increased mortality (log-rank *P* = 0.008) and reduced valve durability.^10^ A 10 cm² increase in annular area can significantly reduce the risk of PPM [OR: 0.94 (0.91–0.97) *P* < 0.0001].^11^ The SMART trial compared self-expanding (Evolut PRO/FX) and balloon-expandable (SAPIEN 3/Ultra) valves in patients with small annuli (<430 mm²). Although balloon-expanding valves demonstrated non-inferiority in clinical endpoints at two years, they were associated with higher rates of valve dysfunction (48.4% vs. 12.5%, *P* < 0.001) and higher gradients. However, data regarding TAVI-in-TAVI procedures in this subset of patients remain extremely limited, preventing to draw definitive conclusions on this topic. Eventually, decisions need to be taken on a case-to-case basis, balancing the risk of anulus rupture vs. higher residual post-procedural gradient and the risk of future bioprosthesis degeneration after TAVI redo. Further studies are needed to address the balance between procedural safety and results in this complex setting.

## Conclusion

This case underscores the complexity of managing TAVI-in-TAVI in patients with small annuli and Morquio syndrome. Optimal outcomes require a detailed anatomical and functional assessment, careful device selection, and coordinated multidisciplinary collaboration.

## Data Availability

The data underlying this article are not publicly available due to patient privacy concerns but can be shared on reasonable request to the corresponding author.

## Lead author biography

I graduated in Medicine from Cambridge University (UK), then obtained a cardiology specialization and PhD at Sapienza University of Rome. I further completed a II Level Master’s degree at Sant’Anna School of Advanced Studies, Pisa, focused on percutaneous interventional treatment of structural heart disease. I am currently a Consultant Interventional Cardiologist at San Camillo Hospital, Rome. In my practice, I perform coronary interventions and percutaneous structural heart disease procedures, including transcatheter aortic valve implantation. My professional commitment centres on innovation in structural and coronary interventions, combining clinical practice with continuous research and academic collaboration to enhance patient outcomes.

## Notes

**Consent:** Written informed consent for publication was obtained from the patient in accordance with COPE guidelines.
